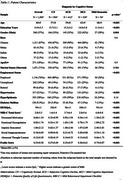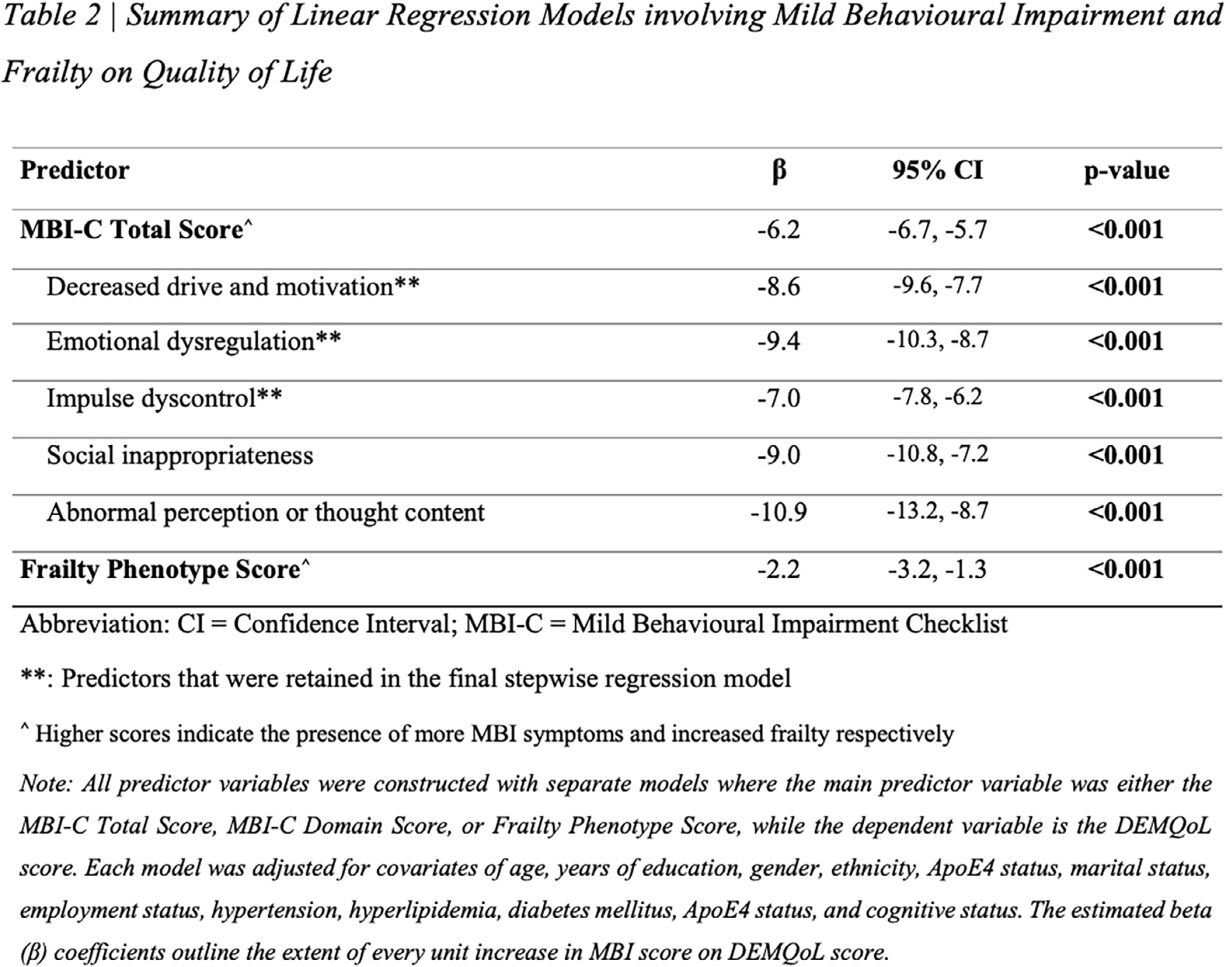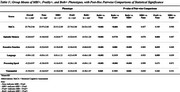# Double Burden of Mild Behavioural Impairment and Frailty – Implications for Quality of Life and Cognition in a Southeast Asian Cohort

**DOI:** 10.1002/alz70861_108032

**Published:** 2025-12-23

**Authors:** Ming Hui, Colin Goh, Yi Jin Leow, Pricilia Tanoto, Nagaendran Kandiah

**Affiliations:** ^1^ Lee Kong Chian School of Medicine, Singapore, Singapore, Singapore Singapore; ^2^ Dementia Research Centre (Singapore), Lee Kong Chian School of Medicine, Nanyang Technological University, Singapore Singapore; ^3^ Lee Kong Chian School of Medicine, Nanyang Technological University, Singapore Singapore; ^4^ Lee Kong Chian School of Medicine, Singapore, Singapore Singapore; ^5^ Neuroscience and Mental Health Programme, Lee Kong Chian School of Medicine, Nanyang Technological University, Singapore Singapore; ^6^ National Healthcare Group, Singapore Singapore; ^7^ Duke‐NUS Medical School, National University of Singapore, Singapore Singapore

## Abstract

**Background:**

While the independent associations of Mild Behavioural Impairment (MBI) and frailty with Quality of Life (QoL) and cognitive outcomes have been independently studied, their combined effects remain less understood, particularly in Southeast Asian populations.

**Method:**

Cross‐sectional analysis of 1,500 participants from the Biomarkers and Cognition Study, Singapore (BIOCIS), including cognitively normal, subjective cognitive decline, mild cognitive impairment and mild dementia.

Behavioural symptoms were assessed using the Mild Behavioural Impairment Checklist (MBI‐C), with a cut‐off score ≥5.5 indicating significant symptoms (MBI+). Frailty was defined by the Fried Frailty Phenotype, with ≥1 criterion indicating frailty (Frail+). Quality of Life (QoL) was measured via the Dementia Quality of Life Questionnaire (DEMQoL), global cognition with the Montreal Cognitive Assessment (MoCA), and domain‐specific cognition (episodic memory, executive function, language, processing speed, visuospatial) using aggregated z‐scores from standardised tests. Multiple linear regression models examined the effects of MBI‐C Total and domain scores, as well as frailty, on DEMQoL, controlling for age, gender, ethnicity, years of education, ApoE4 status, marital status, employment status, hypertension, hyperlipidaemia, diabetes, and cognitive status. Stepwise regression was used to explore combined effects of MBI‐C domains and frailty. Participants were cross‐classified into four MBI/frailty groups, and cognitive outcomes (MoCA and domain‐specific z‐scores) were compared using Analysis of Covariance (ANCOVA) with Tukey Post‐Hoc tests.

**Result:**

Higher MBI‐C Score correlated with poorer DEMQoL (β=‐6.2; *p* <0.001). MBI‐C‐domains emotional dysregulation (β=‐9.4; *p* <0.001), impulsivity (β=‐7.0; *p* <0.001), and apathy (β=‐8.6; *p* <0.001) were associated with poorer QoL than frailty (β=‐2.2; *p* <0.001). Individuals with both MBI and Frailty demonstrated the poorest cognitive performance across all domains (*p* <0.001) except visuospatial (*p* <0.05).

**Conclusion:**

Emotional dysregulation, impulsivity, and apathy are associated with substantially poorer QoL. Co‐occurrence of MBI and frailty are associated with a greater cognitive decline than either condition alone.